# Influence of Indoor Temperature Exposure on Emergency Department Visits Due to Infectious and Non-Infectious Respiratory Diseases for Older People

**DOI:** 10.3390/ijerph18105273

**Published:** 2021-05-15

**Authors:** Chien-Cheng Jung, Nai-Tzu Chen, Ying-Fang Hsia, Nai-Yun Hsu, Huey-Jen Su

**Affiliations:** 1 Department of Public Health, China Medical University, Taichung City 406060, Taiwan; ccjung@mail.cmu.edu.tw; 2 Research Center of Environmental Trace Toxic Substances, National Cheng Kung University, Tainan City 70403, Taiwan; naitzu@gmail.com; 3 Big Data Center, China Medical University Hospital, Taichung City 404332, Taiwan; ivyliveshe@gmail.com; 4 Department of Environmental and Occupational Health, College of Medicine, National Cheng Kung University, Tainan City 70403, Taiwan; boni.hsu@gmail.com

**Keywords:** indoor temperature, older people, respiratory diseases, cumulative degree hour

## Abstract

Previous studies have demonstrated that outdoor temperature exposure was an important risk factor for respiratory diseases. However, no study investigates the effect of indoor temperature exposure on respiratory diseases and further assesses cumulative effect. The objective of this study is to study the cumulative effect of indoor temperature exposure on emergency department visits due to infectious (IRD) and non-infectious (NIRD) respiratory diseases among older adults. Subjects were collected from the Longitudinal Health Insurance Database in Taiwan. The cumulative degree hours (CDHs) was used to assess the cumulative effect of indoor temperature exposure. A distributed lag nonlinear model with quasi-Poisson function was used to analyze the association between CDHs and emergency department visits due to IRD and NIRD. For IRD, there was a significant risk at 27, 28, 29, 30, and 31 °C when the CDHs exceeded 69, 40, 14, 5, and 1 during the cooling season (May to October), respectively, and at 19, 20, 21, 22, and 23 °C when the CDHs exceeded 8, 1, 1, 35, and 62 during the heating season (November to April), respectively. For NIRD, there was a significant risk at 19, 20, 21, 22, and 23 °C when the CDHs exceeded 1, 1, 16, 36, and 52 during the heating season, respectively; the CDHs at 1 was only associated with the NIRD at 31 °C during the cooling season. Our data also indicated that the CDHs was lower among men than women. We conclude that the cumulative effects of indoor temperature exposure should be considered to reduce IRD risk in both cooling and heating seasons and NIRD risk in heating season and the cumulative effect on different gender.

## 1. Introduction

The extreme temperature has critical effects on human health. Numerous studies have investigated the risk of respiratory diseases associated with extreme outdoor temperature exposure [1,2,3] and have demonstrated that extreme temperature change is an important risk factor. Some studies have further revealed that temperature change is associated with mortality from infectious respiratory diseases (IRD) [4,5]. Temperature change could also affect the concentrations of inflammatory markers [6,7], such as C-reactive protein, fibrinogen, and interleukin-6 (IL-6), which induces non-infectious respiratory diseases (NIRD) [8,9,10], such as asthma and chronic obstructive pulmonary disease (COPD). Thus, the outdoor temperature change could also be a risk factor for NIRD.

However, on average, >85% of the day is spent indoors [11]. Due to global warming, the frequency of people-to-people contact significantly increases or changes human behaviors indoors (such as ventilation system usage) [12]. Thus, the possibility of person-to-person transmission of infection increases indoors, which increases IRD risk. Furthermore, the temperature is an essential risk factor for NIRD, and because people spend most of their time indoors, the indoor temperature may be a more decisive factor than the outdoor temperature for NIRD risk. According to our knowledge, only one study investigated the association between indoor temperature exposure and respiratory disease risk [13], but that study did not investigate the association between temperature exposure and IRD and NIRD.

Additionally, Sexton and Hattis reported that exposure frequency, time, and level are key factors influencing the association between environmental factor exposure and disease risk [14]. Byrne’s study measured the body’s temperature and the heart rate and found that the area-specific body temperature and exposure time changed significantly over time (30 min, 60 min, 90 min, etc.) [15]. They suggested that the cumulative effect of temperature exposure was a more suitable index for describing the health effect than single-point temperature change. Thus, some studies have used heating and cooling degree hours for assessing the effects of energy consumption indoors on thermal comfort [16,17]. Our previous study also used cumulative degree hours (CDHs) to investigate the cumulative effects of indoor temperature exposure on cardiovascular diseases [18]. However, respiratory disease was one of the top 10 leading causes of death in Taiwan in 2019 (https://www.mohw.gov.tw/cp-16-54482-1.html, accessed on 16 June 2020), but no study investigated the cumulative effect of indoor temperature exposure on the risks of IRD and NIRD.

United Nations report indicated that the population aged > 60 years in 2050 will be twice that in 2017 [19]. Moreover, Taiwan’s Ministry of the Interior [20] indicated that the population aged > 65 years was 7% in 1993 and will be 20% in 2026. A study found that older adults spend more time indoors than younger populations do (<59 years old) [11]. One study also identified that older people have lower ability than younger people to adapt to the temperature change [21]. Thus, we cannot ignore the effect of indoor temperature exposure on older adults. Additionally, men and women have different thermoregulation abilities [7], and one study demonstrated differences in sweat loss based on gender [22]. Therefore, investigating the association between indoor temperature exposure and respiratory diseases in different gender among older adults is necessary.

The objective of this study was to estimate the cumulative effects of indoor temperature exposure by using CDHs on emergency department visits for IRD and NIRD among older adults. We also examined individually investigated the association between IRD or NIRD and CDHs on men and women for understanding indoor temperature exposure effect on different gender. This information will provide more complete guidance to decision-makers formulating polices related to public health and further consider the effect for different gender.

## 2. Material and Method

### 2.1. Study Participants

An ecological study design was conducted to analyze the association between the cumulative effect of indoor temperature exposure by using CDHs and emergency department visits for IRD and NIRD. The subjects were collected from the Longitudinal Health Insurance Database (LHID) of the National Health Insurance (NHI) program from 2006 to 2014. The LHID was derived from Taiwan’s NIH program. There were 2,000,118 participants that were randomly selected from the year 2006 registry of beneficiaries (N = 25.68 million) under the NHI program from 317 townships on the island of Taiwan. In this study, we included the population older than 65 years from LHID, a total of 231,282 participants. Among 231,282 participants, from 2006 to 2014, there were 31,755 (based on the International Classification of Diseases, Ninth Revision (ICD-9): 460 to 466 and 480 to 487) and 27,435 (ICD-9: 470 to 478 and 490 to 519) participants who got IRD and NIRD, respectively, who were included in this study.

Since 1995, the NHI program was initiated to finance health care for all citizens in Taiwan. The NHI program contains more than 99% of the entire population of Taiwan. The NHI program has universal health insurance coverage, a single-payer system with the government as the sole insurer and payer, comprehensive benefits, unrestricted choice of physician and medical institution, and a variety of institutional providers well distributed throughout the country. Taiwanese researchers use an anonymous identification number to link each registry data and related medical information for academic purposes. Recently, some studies also used LHID to investigate public health issues [23,24,25].

### 2.2. Indoor Temperature Estimation

Before calculating the CDHs, we estimated prediction models of hourly indoor temperature for Taiwan’s households. The detailed processes and materials of the indoor temperature prediction model are reported in our previous study [18]. Briefly, the hourly levels of indoor and outdoor weather data, normalized difference vegetation index (NDVI), building characteristics, occupants’ behavior, and electricity consumption were collected from 2012 to 2015 in Taiwan from 30 households. After we collected these data, we used a mixed effect model to estimate a prediction model of hourly indoor temperature. The weather conditions differ by month, so we also estimated the prediction model of indoor temperature for each month.

We further calculated the hourly indoor temperature from 2006 to 2014 (match the years to emergency department visits due to IRD and NIRD from LHID) based on the prediction models of hourly indoor temperature as mentioned previously for Taiwan households for each month. To calculate hourly indoor temperature, we collected weather (indoor/outdoor temperature (°C), indoor/outdoor relative humidity (%), atmospheric pressure (mmHg), global solar radiation (Million-Joule/m^2^), wind speed (m/s), and wind direction (°), land surface temperature (°C), NDVI, electricity consumption (kilowatt-hour/day), and building characteristics (building structure type: stone or reinforced concrete, building age (year), building level (floor)) between 2006 and 2014 from Taiwan’s Central Weather Bureau, National Aeronautics and Space Administration (NASA, Washington, DC, USA), NASA, Taiwan power company, and statistical report of Ministry of the Interior in Taiwan, respectively. The detailed variables of the prediction model of hourly indoor temperature were presented in our previous study [18].

### 2.3. Cumulative Degree Hour Calculation

After we calculated the hourly indoor temperature from 2006 to 2014, the indoor temperature was used to calculate CDHs by using Equations (1) and (2) for cooling and heating degree hours in hot (May to October) and cold (November to April) seasons [18], respectively:

(1) $$\mathrm{Cooling}\;\mathrm{degree}\;\mathrm{hours}=\sum_{i=1}^{N}\left(T_{i}-T_{b}\right)$$

(2) $$\mathrm{Heating}\;\mathrm{degree}\;\mathrm{hours}=\sum_{i=1}^{N}\left(T_{b}-T_{i}\right)$$

where *T_i_* is the indoor temperature from prediction models of hourly indoor temperature for Taiwan’s households, *T_b_* is the threshold, and *N* is the hours in a day. The *T_i_* was calculated from the prediction models of hourly indoor temperature. A previous study [26] found that the risk of emergency department visits due to respiratory diseases (included IRD and NIRD) was significant when the indoor temperatures ranged from 27 °C to 31 °C during the cooling season (May to October) and 19 °C to 23 °C during the heating season (November to April). Therefore, the thresholds were 27 °C and 23 °C of both cooling and heating seasons, respectively. Moreover, we assumed that the study participants remained indoors all day, because previous studies indicated that older people spend most of their time indoors [11].

### 2.4. Data Analysis for CDHs and Emergency Department Visit

A distributed lag nonlinear model with a quasi-Poisson function was applied to assess the relationship between CDHs and emergency department visits for IRD or NIRD until CDHs were associated with emergency department visit risk for IRD or NIRD. The quasi-Poisson function is a generation of Poisson regression. Studies have indicated that air pollution is also one of the principal factors that influence the risk of respiratory diseases [27,28]; therefore, we collected air pollutant concentrations (NO_x_, PM_2.5_, and O_3_, we calculated daily average concentration from hourly concentrations) from Taiwan’s Environmental Protection Administration from 2006 to 2014 to adjust the association between CDHs and outcomes. We adjusted the day of the week (DOW) because the number of emergency department visits were different in different study days; during the holiday, the number of emergency department visits could increase because the outpatient was closed [29], and this study also adjusted the holiday. Equation (3) shows the analysis model:

(3) $$\begin{array}{l} \log\left(E\left(Y\right)\right)=\beta_{0}+\overset{7}{\underset{t=0}{\sum }}NS\left(CDH_{t},DF,\;5;lag,\;7\right)+\overset{1}{\underset{t=0}{\sum }}Lin\left(PM_{2.5\;t}\right)+\overset{1}{\underset{t=0}{\sum }}Lin\left(O_{3\;t}\right)+\overset{1}{\underset{t=0}{\sum }}Lin\left(NO_{x\;t}\right)\\ \;\;\;\;\;\;+NS\;\left(\mathrm{Time},\;\frac{7}{year}\right)+DOW+Holiday \end{array}$$

*Y*: emergency department visits due to respiratory diseases (IRD or NIRD); *NS*: nature cubic spline; *CDH*: cumulative degree hour; *DF*: degree of freedom; lag: lag day; time: node; *DOW*: day of the week. SAS 9.4 software (v9.4, SAS Institute Inc., Cary, NC, USA) was used to analyze all data, statistical significance was set at *p* < 0.05. 

The study was approved by the Human Research Ethics Committee of National Cheng-Kung University Hospital (HREC (Exempt) NO. 106-001).

## 3. Results

### 3.1. Cases in Emergency Department Visit Due to Respiratory Diseases

The CDHs of indoor temperatures are presented in Table 1. The CDHs were 32.9 ± 25.0, 16.8 ± 17.2, 6.2 ± 9.2, 1.3 ± 3.3, and 0.1 ± 0.5 at 27, 28, 29, 30, and 31 °C during the cooling season, respectively; the CDHs were 29.3 ± 35.8, 17.7 ± 27.1, 9.3 ± 18.6, 4.0 ± 11.2, and 1.3 ± 5.7 at 23, 22, 21, 20, and 19 °C during the heating season, respectively.

The summary of emergency department visits due to IRD and NIRD in different subgroups are presented in Table 2. Our data showed that the patients with IRD and NIRD were 14,371 and 12,417 during the cooling season, respectively; the patients with IRD and NIRD were 17,384 and 15,018 during the heating season, respectively. Moreover, 8671 and 8393 IRD and NIRD emergency department admitters were men and 5700 and 4024 IRD and NIRD were women during the cooling season; 10,144 and 10,000 IRD and NIRD emergency department admitters were men and 7240 and 5018 IRD and NIRD were women during the heating season. As expected, the proportions of IRD and NIRD were higher in men than in women. In Taiwan, the rates of tobacco use and alcohol consumption are higher among men than among women based on Ministry of Health and Welfare data (https://reurl.cc/OX9zq7, accessed on 5 January 2021). Both smoking and alcohol consumption are major risk factors for IRD and NIRD [30,31,32], which explained the higher proportions of IRD and NIRD among men.

### 3.2. Cumulative Effects of Indoor Temperature Exposure on Respiratory Diseases

Table 3 presents the CDHs for IRD and NIRD for various indoor temperatures. The data indicate that the risk of emergency department visits due to IRD and CDH decreased with the increase in the indoor temperature (*p* < 0.05) during the cooling season. During the heating season, the risk of emergency department visits due to IRD and CDH decreased with the decrease in indoor temperature (*p* < 0.05).

For NIRD, a significant risk of emergency department visits only occurred at 31 °C during the cooling season when the CDHs exceeded 1 (*p* < 0.05). During the heating season, a significant risk of emergency department visits due to NIRD occurred, and the CDHs decreased with the decrease in indoor temperature (*p* < 0.05).

We further analyzed the differences between gender regarding the cumulative effects of indoor temperature exposure, as displayed in Table 4 and Table 5. Among men, a significant risk of emergency department visits due to IRD occurred, the CDHs decreased with the increase in indoor temperature (*p* < 0.05) during the cooling season, and decreased with the decrease in indoor temperature during the heating season. Among women, a significant risk of emergency department visits due to IRD occurred, the CDHs decreased with the increase in indoor temperature (*p* < 0.05) during the cooling season. A significant risk occurred during the heating season when the CDHs exceeded 1, 23, and 96 at 20, 21, and 23 °C (*p* < 0.05), respectively.

In Table 5, during the cooling season, our data indicated that the risk of emergency department visits due to NIRD was significant and the CDHs decreased with the increase in indoor temperature among man; during the heating season, a significant risk of emergency department visits due to NIRD occurred and the CDHs decreased with the decrease in indoor temperature. Among women, the risk of emergency department visits due to NIRD was significant when the CDHs exceeded 73, 45, and 25 at 27 °C, 28 °C, and 29 °C (*p* < 0.05) during the cooling season, respectively; significant risk of emergency department visits due to NIRD occurred and the CDHs decreased with the decrease in indoor temperature during the heating season. Overall, men have fewer CDHs than women in the same indoor temperature ranges.

## 4. Discussion

This is the first study to examine the cumulative effects of indoor temperature exposure on emergency department visits due to IRD and NIRD. Our findings demonstrated that a significant risk of emergency department visits due to IRD occurred when the indoor temperature exceeded 27 °C during the cooling season and the CDHs decreased with increases in indoor temperature; the risk was significant when the indoor temperature was lower than 23 °C during the heating season, and the CDHs decreased with decreasing in indoor temperature. For NIRD, the CDHs was only significantly associated with the NIRD at 31 °C during the cooling season; the risk was significant when the indoor temperature was lower than 23 °C during the heating season. Moreover, the CDHs were lower among men than among women.

Temperature is an important factor influencing IRD risk [12]. According to the LHID data, the major IRD is pneumonia (the occurrence rates of pneumonia, bronchial pneumonia, acute bronchiolitis, and acute upper respiratory tract infection in the cooling and heating seasons were 59%, 8%, 9%, and 12% and 61%, 8%, 8%, and 11%, respectively). Studies have revealed that temperature plays a crucial role in influencing pneumonia risk [33,34], especially in winter. Higher or lower temperatures repress the immune function to resist virus or bacterial infection [9,35,36]. Thus, both high and low temperature exposures increase IRD risk. Occupants should adapt to decrease or avoid indoor transmission, take immediate action when the indoor temperature gradually increases or decreases, or maintain the indoor temperature at an appropriate range to reduce IRD risk.

A study indicated that high temperature exposure increases the levels of inflammatory indicators, which induces respiratory symptoms among patients with respiratory diseases [9]. Moreover, exposure to high temperature increases blood viscosity, which increases mortality in respiratory diseases [8]. Exposure to cold conditions induces tracheal contraction [37] or increases inflammatory indicator concentrations in plasma, such as interleukin-6 (IL-6) or norepinephrine, which induces respiratory diseases [38]. Therefore, both high and low temperature exposures are risk factors for NIRD.

We further analyzed the cumulative effects of indoor temperature exposure on IRD. Overall, the CDHs decreased as the temperature increased and decreased during the cooling and heating seasons, respectively. Global warming influences human behaviors, increases the frequency of people-to-people contact, or influences the survival of infectious contaminants indoors [12], which increases IRD risk. Temperature change also affects the immune system’s capability to resist viral or bacterial infection [35,39], especially at higher or lower temperature conditions. Thus, both high and low temperature exposure and spending most of the time indoors could increase IRD risk. 

Our data showed that the CDHs were associated with NIRD only at 31 °C and < 23 °C during cooling and heating seasons, respectively. Previous study found that temperature change increases the release of inflammatory indexes [40,41], such as interleukin-1, IL-6. or tumor necrosis factor-alpha in the systemic circulation. This affects the vascular endothelium function, coagulation, and fibrinolysis inhibition, which induce respiratory syndrome [42,43]. In this study, the major NIRD was COPD (the occurrence rates of COPD, chronic bronchiolitis, asthma, and other non-respiratory diseases during cooling and heating seasons were 25%, 18%, 22%, and 19% and 26%, 19%, 19%, and 19%, respectively). In winter, cold stress increases the plasma fibrinogen concentrations because of the acute response to the respiratory system [44,45]. Furthermore, one study indicated that emergency department visits for COPD were higher during the cold season than during the hot season in Taiwan [46] as patients with COPD were sensitive to large temperature change [47]. The temperature difference is larger during the cold season than during the hot season in Taiwan. Furthermore, our data indicate that NIRD cases were higher during the heating season than during the cooling heating (Table 2). This may explain the reason for the association of CDHs with NIRD only at 31 °C during the cooling season. Therefore, we should consider the cumulative effects of indoor temperature exposure on NIRD, especially in the heating season; temperature could be an influencing factor for NIRD if the indoor temperature is > 31 °C during the cooling season.

Our data indicated that indoor temperature exposure is significantly associated with IRD and NIRD for both men and women. Emergency department visits due to IRD and NIRD on CDHs were fewer among men than among women. Study indicated that respiratory disease risk is higher among women than among men [48], and the result is not consistent with our data. Human behavior and comorbidities may explain sex-based differences in CDHs. In Taiwan, the rates of tobacco use and alcohol drinking are higher among men than among women based on the Ministry of Health and Welfare data (https://reurl.cc/OX9zq7, accessed on 5 January 2021). Smoking was found to be an important risk factor for asthma and COPD [49,50]. Studies also indicated that smoke exposure impairs immune function and increases infection risk [51,52]. Furthermore, alcohol drinking is one of the risk factors for respiratory diseases [53,54]. Women often keep warm to overcome the temperature change during the heating season, which increases the lag effect of low temperature exposure on respiratory diseases. Men have a high rate of comorbidities and worse treatment outcomes in Taiwan [55]. This may explain the reason for fewer CDHs among men than among women during both cooling and heating seasons.

Table 3 shows that the risk of NIRD was not significant from 27 °C to 30 °C for all subjects and Table 5 indicates that the risk of NIRD was significant from 27 to 30 °C among men and from 27 °C to 29 °C among women. The result implied that the influencing factor were different on the association between high temperature exposure and NIRD risk in different gender. For instance, IL-6 is also a cytokine with heat stress and Yu’s study indicated the level of IL-6 was higher among men than women when they exposure to higher temperature [56], which means men have lower thermoregulation abilities than women during the periods of high temperature exposure. Studies suggested that social condition was also a factor influencing the risk of respiratory diseases and the social condition was different for men and women [57,58]. Men could have higher exposure of high temperature than women due to work outside. This may explain why temperature exposure was associated with NIRD during the cooling season after gender stratified.

Humans have normal physiological responses to the adverse effects of temperature changes. However, humans are also less able to cope with hot or cold exposure when this exposure continues for a long time. In Byrne’s study [15], they measured the association between body temperature and the heart rate and found that the area-specific body temperature and exposure time changed significantly over time (e.g., 30, 60, and 90 min). This study indicated that the cumulative effect of temperature exposure is a more suitable index for describing the health effect than single-point temperature change. The present data revealed that CDH reduces when indoor temperature increases or decreases during the cooling season or the heating season, respectively. Thus, the CDH value cannot be used to represent disease severity, but the CDHs can indicate the amount of time required to engage in protective actions to reduce the risk of diseases when exposed to a hot or cold environment. For example, during the cooling season, people should immediately take protective actions to reduce IRD risk when temperatures reach 31 °C, and a buffer time for implementing protective actions should be implemented when temperatures reach 27 °C.

There were some limitations to this study. First of all, a prediction model of indoor temperature was used to estimate indoor temperature, and thus, our data may not reflect the real exposure situation with respect to skin exposure. However, a study indicated that indoor temperature was associated with skin temperature [59]; thus, we suggested that using the indoor temperature from the prediction model can still to reflect the cumulative effect of exposure to indoor temperatures on the outcome. Second, human activities indoors were highly associated with the variation of indoor temperature. However, human activities were not included in the predictive model. In the future, applying advanced technology to collect the frequency and time of human activities in indoors is necessary. Third, the indoor temperature from the prediction model may not reflect the real exposure situation indoors in Taiwan. Yet, the building characteristics (included age, number of floors, building category, and building material), which the 30 selected households resided were similar to the characteristics of buildings in Taiwan [20]. Therefore, the data from the study households can present the indoor temperature distributions of the majority of households in Taiwan.

## 5. Conclusions

Our data revealed that indoor temperature exposure was associated with IRD and NIRD and the cumulative effects by using CDHs decreased as the indoor temperature increase and decrease during the cooling and heating season, respectively. This reflected that policymakers should consider the cumulative effects of indoor temperature exposure on hot- or cold-related IRD and NIRD. Moreover, our data indicated that the CDHs were lower among men than among women and suggested that policymakers should also consider the cumulative effect for different gender.

## Figures and Tables

**Table 1:** Summary of cumulative degree hours from 2006 to 2014 in different seasons.

Indoor Temperature (°C)	Cumulative Degree Hours	
	Mean ± SD.	Min to Max
**Cooling season (May to October)**		
27	32.9 ± 25.0	0 to 106.9
28	16.8 ± 17.2	0 to 82.9
29	6.2 ± 9.2	0 to 58.9
30	1.3 ± 3.3	0 to 34.9
31	0.1 ± 0.5	0 to 16.5
**Heating season (November to April)**		
19	1.3 ± 5.7	0 to 79.0
20	4.0 ± 11.2	0 to 103.0
21	9.3 ± 18.6	0 to 127.0
22	17.7 ± 27.1	0 to 151.0
23	29.3 ± 35.8	0 to 175.0

**Table 2:** Summary of respiratory disease-related emergency department visits (proportion) from 2006 to 2014 in different seasons.

	Cooling Season (May to October)		Heating Season (November to April)	
	IRD	NIRD	IRD	NIRD
Older adults (≥65 years old)	14,371	12,417	17,384	15,018
Gender				
Man (%)	8671 (60%)	8393 (68%)	10,144 (58%)	10,000 (67%)
Woman (%)	5700 (40%)	4024 (32%)	7240 (42%)	5018 (33%)

IRD: Infectious respiratory diseases; NIRD: Non-infectious respiratory diseases.

**Table 3:** Cooling and heating cumulative degree hours for infectious and non-infectious respiratory disease-related emergency department visits for older adults from 2006 to 2014.

	IRD		NIRD	
Setpoint Temperature (Indoor, °C)	CDH	Relative Risk (95%CI)	CDH	Relative Risk (95%CI)
**Cooling Season (May to October)**				
27	69	1.229 (1.004, 1.508)		NS
28	40	1.180 (1.004, 1.387)		NS
29	14	1.122 (1.001, 1.257)		NS
30	5	1.107 (1.008, 1.219)		NS
31	1	1.213 (1.127, 1.306)	1	1.124 (1.028, 1.229)
**Heating Season (November to April)**				
19	8	1.073 (1.005, 1.330)	1	1.019 (1.004, 1.033)
20	1	1.013 (1.005, 1.022)	1	1.021 (1.012, 1.029)
21	1	1.012 (1.000, 1.012)	16	1.090 (1.001, 1.188)
22	35	1.114 (1.000, 1.241)	36	1.117 (1.001, 1.246)
23	62	1.133 (1.000, 1.285)	52	1.126 (1.001, 1.266)

CDH: Cumulative degree hours; CI: Confidence interval. NS: Not significance.

**Table 4:** Cooling and heating cumulative degree hours for infectious respiratory disease-related emergency department visits for older adults from 2006 to 2014 (gender stratified).

Setpoint Temperature (Indoor, °C)	Man		Woman	
	CDH	Relative Risk (95%CI)	CDH	Relative Risk (95%CI)
**Cooling Season (May to October)**				
27	72	1.299 (1.006, 1.693)	77	1.390 (1.001, 1.931)
28	43	1.240 (1.010, 1.538)	51	1.327 (1.000, 1.760)
29	19	1.167 (1.010, 1.372)	14	1.188 (1.003, 1.406)
30	5	1.146 (1.005, 1.271)	8	1.209 (1.019, 1.434)
31	1	1.185 (1.077, 1.293)	1	1.267 (1.137, 1.411)
**Heating Season (November to April)**				
19	1	1.026 (1.007, 1.040)		NS
20	1	1.018 (1.004, 1.025)	1	1.012 (1.000, 1.023)
21	23	1.127 (1.004, 1.260)	23	1.151 (1.001, 1.323)
22	36	1.116 (1.002, 1.303)	96	1.298 (1.002, 1.682)
23	60	1.156 (1.001, 1.351)		NS

CDH: Cumulative degree hours; CI: Confidence interval. NS: Not significance.

**Table 5:** Cooling and heating cumulative degree hours for non-infectious respiratory disease-related emergency department visits for older adults from 2006 to 2014 (gender stratified).

Setpoint Temperature (Indoor, °C)	Man		Woman	
	CDH	Relative Risk (95%CI)	CDH	Relative Risk (95%CI)
**Cooling season (May to October)**				
27	44	1.276 (1.005, 1.620)	73	1.586 (1.005, 2.504)
28	20	1.199 (1.000, 1.437)	45	1.459 (1.001, 2.126)
29	1	1.033 (1.015, 1.052)	25	1.366 (1.010, 1.848)
30	9	1.198 (1.005, 1.428)		NS
31	1	1.174 (1.046, 1.317)		NS
**Heating season (November to April)**				
19		NS	1	1.026 (1.003, 1.050)
20	1	1.019 (1.009, 1.029)	1	1.024 (1.010, 1.038)
21	1	1.007 (1.000, 1.015)	32	1.199 (1.002, 1.435)
22	38	1.142 (1.002, 1.301)	51	1.222 (1.005, 1.486)
23	61	1.162 (1.003, 1.347)	70	1.238 (1.000, 1.532)

CDH: Cumulative degree hours; CI: Confidence interval. NS: Not significance.

## Data Availability

The data presented in this study are available on reasonable request from the corresponding author.
